# Serum angiopoietin-2 concentrations of post-PCI are correlated with the parameters of renal function in patients with coronary artery disease

**DOI:** 10.1097/MD.0000000000013960

**Published:** 2019-01-04

**Authors:** Wen Jian, Lang Li, Xiao-Min Wei, Jia-Hui Guan, Guo-Liang Yang, Chun Gui

**Affiliations:** a Department of Cardiology, The First Affiliated Hospital of Guangxi Medical University; b Guangxi Key Laboratory Base of Precision Medicine in Cardio-Cerebrovascular Diseases Control and Prevention; c Guangxi Clinical Research Center for Cardio-Cerebrovascular Diseases, Nanning; d Department of Cardiology, Gongren Hospital of Wuzhou, Wuzhou; e Department of Respiratory Medicine, The First Affiliated Hospital of Guangxi Medical University, Nanning, Guangxi, People's Republic of China.

**Keywords:** angiopoietin-2, biomarker, chronic kidney disease, coronary artery disease, percutaneous coronary intervention

## Abstract

Patients with coronary artery disease (CAD) frequently have comorbidity of chronic kidney disease (CKD). Their renal function may deteriorate because of the use of contrast agent after percutaneous coronary intervention (PCI). Angiopoietin-2 (Ang-2), which is highly expressed in the site of angiogenesis, plays an important role in both CAD and CKD. This study aimed to investigate the relation of serum Ang-2 concentrations with the renal function after PCI.

This study enrolled 57 patients with CAD undergoing PCI. Blood samples for Ang-2 were collected in the first morning after admission and within 24 to 48 h after PCI. The parameters of renal function (serum creatinine, cystatin C and eGFR) were tested on the first day after admission and within 72 h after PCI.

Overall, serum Ang-2 levels of post-PCI were significantly lower than those of pre-PCI [median, 1733 (IQR, 1100–2568) vs median, 2523 (IQR, 1702–3640) pg/mL; *P* < .001]. However, in patients with CKD (eGFR < 60 mL/min/1.73 m^2^), there was no significant difference between serum Ang-2 levels of post-PCI and those of pre-PCI [median, 2851 (IQR, 1720–4286) vs. median, 2492 (IQR, 1434–4994) pg/mL; *P* = .925]. In addition, serum Ang-2 levels of post-PCI, but not pre-PCI, were significantly correlated with the post-PCI parameters of renal function.

Serum Ang-2 concentrations of post-PCI are closely related to renal function in patients with CAD. It may have potential to be the early biomarker of contrast-induced nephropathy in the future.

## 1 Introduction

Coronary artery disease (CAD) is the most common cause of death globally. Limitation of blood flow to the heart can cause ischemia and dysfunction of the myocardial cells. Angiogenesis, which occurs in the site of anoxic tissue, can lead to the formation of new blood vessels and is crucial for the self-compensation in response to ischemia from coronary stenosis or occlusion.^[1]^ Several growth factors are closely associated with angiogenesis such as angiopoietin-1 (Ang-1), angiopoietin-2 (Ang-2), and vascular endothelial growth factor. The angiopoietin/Tie-2 ligand receptor system is an important regulator of the vascular integrity and angiogenesis.^[2]^ Binding of the agonist Ang-1 to the Tie2 receptor promotes vascular stabilization,^[3]^ whereas Ang-2 inhibits binding of Ang-1 to Tie2 disrupting the Tie2 signaling. High level of Ang-2 can result in the destabilization of endothelial cell junctions to enhance new vessel branching and sprouting.^[4]^ Ang-2 is stored in the Weibel-Palade bodies within the endothelial cells and can be rapidly released upon various stimuli.^[5]^ Ang-2 expression is significantly up-regulated in ischemic or necrotic myocardium.^[1,6]^ Clinical studies have indicated that peripheral blood Ang-2 concentrations are elevated in patients with CAD and are associated with the severity of coronary artery stenosis.^[7,8]^

Of note, because of lots of common risk factors, a great number of patients with CAD have varying degrees of chronic kidney disease (CKD). The heart failure caused by CAD can also lead to renal insufficiency. In addition, it is reported that CKD patients have higher risk of developing cardiovascular disease (CVD), which indicate the highly interrelated feature of them.^[9,10]^ Ang-2, besides its role in angiogenesis, also controls the vascular inflammation^[11]^ and plays an important role in CKD. Pathophysiological characteristics of CKD, such as hypoxia, tumor necrosis factor-α (TNF-α), and reactive oxygen species (ROS), can lead to endothelial Ang-2 secretion;^[11–13]^ Serum Ang-2 levels increase with progression of CKD and are closely related to renal function.^[14]^ Besides, serum angiopoietin-2 is associated with albuminuria and markers of systemic microinflammation in CKD patients.^[15]^ Even in the general population, after exclusion of subjects with hypertension or diabetes mellitus, serum Ang-2 concentrations are also highly associated with the parameters of renal function.^[16]^ In vivo, glomerular Ang-2 is increased in glomerulonephritis.^[17]^ In renal ischemia/reperfusion injury, Ang-2 is highly expressed and involved in renal microvascular remodeling and development of fibrosis.^[18]^

Our previous study showed that serum Ang-2 concentrations are elevated in patients with CAD and decrease significantly after percutaneous coronary intervention (PCI).^[7]^ However, there are still some patients whose Ang-2 levels decrease slightly or even increase after PCI, which exact mechanisms remain unclear. Given the fact that lots of patients with CAD have a certain degree of renal insufficiency, and their renal functions may deteriorate during the process of PCI because of the use of contrast agent. Therefore, in addition to the relief of myocardial ischemia, the status of renal function may also influence the serum Ang-2 level of post-PCI. Based on the prior data, we hypothesized that Ang-2 level of post-PCI may have close relations with renal function.

Given the above information, this study aimed to determine whether serum Ang-2 levels of post-PCI are correlated with renal-function parameters [serum creatinine (Scr), cystatin C (CysC) and estimated glomerular filtration rate (eGFR)] of pre-PCI or post-PCI.

## 2 Methods

### 2.1 Patients and study design

This study enrolled 57 patients with CAD undergoing PCI. The following exclusion criteria were defined: ST-elevation myocardial infarction (STEMI), valvular heart disease, systemic infectious diseases, tumor and autoimmune diseases. The study protocol was reviewed and approved by the Human Research Ethics Committee of the First Affiliated Hospital of Guangxi Medical University, China. Written informed consents were obtained from all patients. The clinical data of patients were collected on the first day after admission. Standard medical treatments were given to all patients for their clinical conditions. All patients were used of the drug-eluting stent, and the dosage of contrast agent was used as needed.

### 2.2 Laboratory measurements

The result of Scr and CysC were collected based on the data of the routine laboratory test of the hospital on the first day after admission and within 72 h after PCI. eGFR was calculated using CKD-EPI_**(Scr-CysC)**_ equation.^[19]^ Venous blood samples for Ang-2 were collected in the first morning after admission and within 24 to 48 h after PCI. The samples were kept without an anticoagulant for 0.5 h at room temperature, and then centrifuged at 5000 r/min for 10 min. The serum supernatant was removed and stored at −80°C until it was used for analysis. According to the manufacturer's instructions of enzyme-linked immunosorbent assay kits (RayBiotech, Inc, Norcross, GA), Ang-2 concentrations were measured in duplicate and averaged.

### 2.3 Statistical analysis

Continuous variables are presented as mean ± SD or median (IQR, interquartile range), and were compared using the Student *t* test or the Mann-Whitney *U* test, as appropriate. Categorical variables were compared using the chi-square or the Fisher exact test. The distribution of Ang-2, which was verified by Kolmogorov–Smirnov test, was skewed. To determine whether there was difference in serum Ang-2 levels before and after PCI, the Wilcoxon signed-rank test was used. The correlations of serum Ang-2 levels with the renal-function parameters were analyzed using the Spearman rank correlation test. *P* values < .05 were considered statistically significant. The statistical analysis was conducted using SPSS, version 19.

## 3 Results

### 3.1 Patient characteristics

The baseline characteristics of patients with CAD are summarized in Table 1. Based on the eGFR levels of pre-PCI, the patients were divided into CKD group (eGFR < 60 mL/min/1.73 m^2^) and non-CKD group (eGFR ≥60 ml/min/1.73 m^2^). No significant difference was found between the groups in terms of gender, BMI, diabetes, hyperlipidemia, smoking, left ventricular ejection fraction, myocardial enzyme, lesion vessel features, treated vessels numbers and all the medications at admission. However, the CKD patients were characterized by an older age and higher blood pressure. Of note, the patients with CKD had similar Ang-2 levels of pre-PCI [median, 2492 (IQR, 1434–4994) vs median, 2523 (IQR, 1840–3232) pg/mL; *P* = .911], but significant higher Ang-2 levels of post-PCI [median, 2851 (IQR, 1720–4286) vs median, 1552 (IQR, 1043–2438) pg/mL; *P* = .013] compared with the non-CKD patients.

**Table 1:**
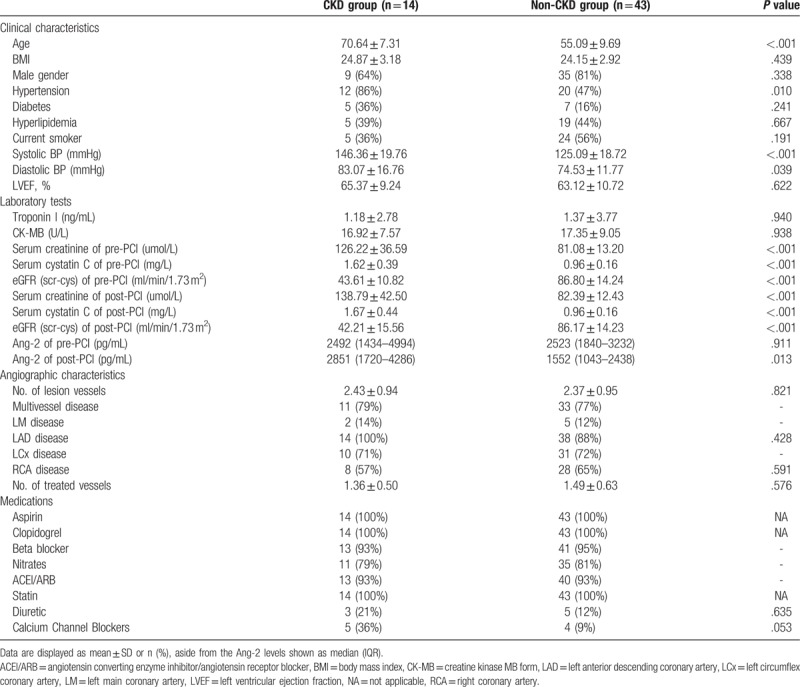
Baseline characteristics of CKD group and non-CKD group.

### 3.2 Changes in serum angiopoietin-2 levels after PCI

**Figure 1:**
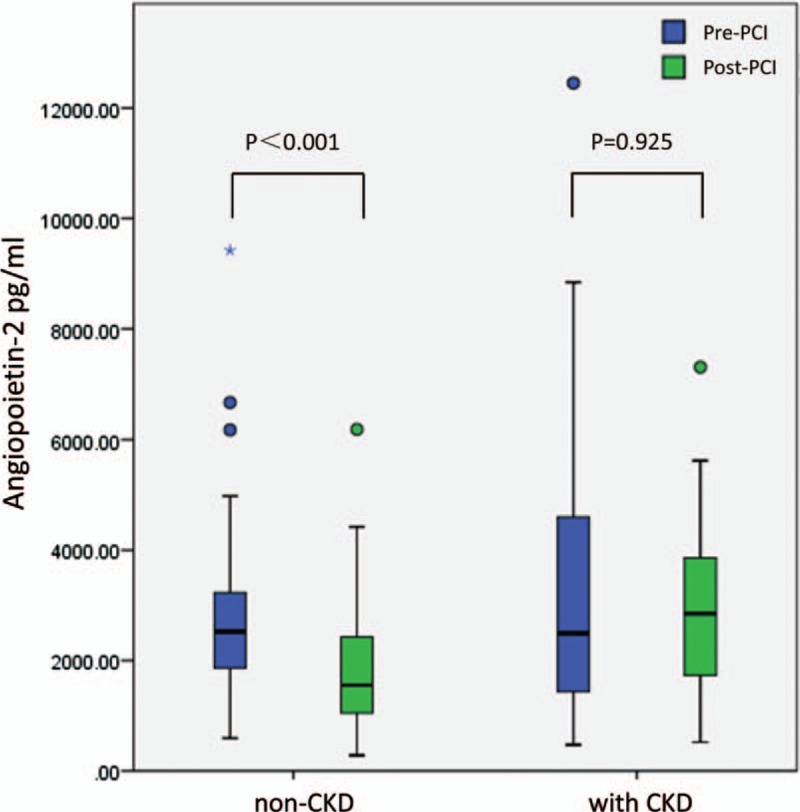
The change of serum Ang-2 levels after PCI in non-CKD group or CKD group.

There was no clinical evidence of interventional complications during the process of PCI. Overall, serum Ang-2 levels of post-PCI were significantly lower than those of pre-PCI [median, 1733 (IQR, 1100–2568) vs. median, 2523 (IQR, 1702–3640) pg/mL; *P* < .001]. However, as shown in Figure 1, in patients with CKD, there was no significant difference between serum Ang-2 levels of post-PCI and those of pre-PCI [median, 2851 (IQR, 1720–4286) vs median, 2492 (IQR, 1434–4994) pg/mL; *P* = .925].

### 3.3 Correlation of the serum Ang-2 with the parameters of renal function

**Figure 2:**
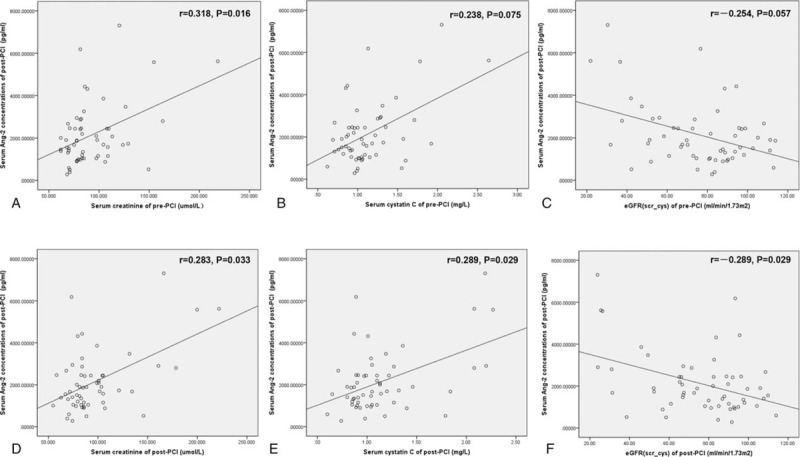
Correlation of serum Ang-2 levels of post-PCI with the renal-function parameters of pre- or post-PCI (Spearman rank correlation test).

By Spearman rank correlation test, serum Ang-2 levels of pre-PCI were unrelated to any parameter of renal function (data were not shown). However, as can be seen from Figure 2, serum Ang-2 levels of post-PCI were significantly correlated with the renal-function parameters of post-PCI (Scr, r = 0.283, *P* = .033; CysC, r = 0.289, *P* = .029; eGFR, r = −0.289, *P* = .029). However, for the renal-function parameters of pre-PCI, only Scr levels were found to be correlated with serum Ang-2 levels of post-PCI (r = 0.318, *P* = .016).

## 4 Discussion

The major findings of the present study are:

(1) serum Ang-2 levels of post-PCI are significantly correlated with the renal-function parameters of post-PCI.

(2) Serum Ang-2 levels in non-CKD patients decrease significantly after PCI, while in CKD patients, serum angiopoietin-2 levels have no significant change after PCI.

This study indicated the complexity of Ang-2 in CAD patients, especially the ones with renal dysfunction. Based on all the evidence mentioned above, we can deduce that both ischemic myocardium and dysfunctional kidney contribute to the increased serum concentrations of Ang-2 in patients with CAD. The ischemic myocardium is probably dominant factor influencing the Ang-2 levels of pre-PCI, which have been demonstrated only to be correlated with severity of coronary artery stenosis.^[7,8]^ After the relief of myocardial ischemia by PCI and the different extent of renal injury by contrast agent, the status of renal function may turn to be major influence factor of serum Ang-2 levels of post-PCI. Before the study, we assumed that only the renal-function parameters of post-PCI, not pre-PCI, were likely to have association with Ang-2 of post-PCI. Probably because of the small sample size and the low incidence of contrast-induced nephropathy (CIN), we still observed a correlation between the Scr of pre-PCI and the Ang-2 of post-PCI. Further study which enlarges the sample size will make the results more accurate.

The serum Scr levels, which have been conventionally used for the diagnosis of CIN, are relatively insensitive to the rapid GFR changes seen in acute kidney injury (AKI). Scr levels start to rise only when a significant (>50%) number of nephrons are damaged and typically take 2 to 3 days to reach the diagnostic threshold, thus reducing its value as a marker of AKI. In addition, Scr levels are also influenced by lots of non-renal factors.^[20]^ Therefore, alternative biomarkers have been sought recently. Cystatin C, a low molecular-weight protein produced by all nucleated cells, is freely filtered by the glomerulus and fully reabsorbed and catabolized by the proximal tubules. Serum cystatin C levels are less affected by non-renal factors, and have been demonstrated to be a more accurate early biomarker of GFR reduction compared with Scr.^[21]^ It has been found that elevated serum cystatin C level is an independent predictor of CIN.^[22]^ Neutrophil gelatinase-associated lipocalin (NGAL), a 25-kDa protein covalently bound to neutrophil gelatinase, has been recognized as the earliest biomarker of AKI for now. The serum NGAL levels can rise within 2 h after AKI and has been demonstrated to be highly correlated with the subsequent increased Scr levels.^[23]^ In addition, its prognostic role for adverse cardiovascular events is also of great value.^[24]^ But, to date, we haven’t found any data on the role of Ang-2 in CIN after PCI.

In patients with acute pancreatitis, increased serum Ang-2 concentrations are correlated with deteriorated renal function in the early phase, which indicates the potential role of Ang-2 as a predictor of acute pancreatic-renal syndrome.^[25]^ In critically ill patients, higher plasma Ang-2 concentrations are associated with higher risk of AKI.^[26]^ In acute myocardial infarction, the Ang-2 levels of day 1 can predict the development of AKI, and its levels are being a rising tendency over time in patients with AKI compared with those without.^[27,28]^ Therefore, given the strong link to the renal function, Ang-2 of post-PCI may also have potential to be the early biomarker of CIN. Ang-2 is stored in endothelial Weibel-Palade bodies and can be rapidly released through endothelial activation.^[5]^ The only known inhibitor of Ang-2 exocytosis from Weibel-Palade bodies is nitric oxide,^[29]^ which has decreased availability in patients with CIN. Besides, the vasoconstriction induced by contrast agent can lead to the reduction in renal blood flow, which results in the ischemia of medulla and release of ROS.^[20]^ These pathophysiology mechanisms may make the serum Ang-2 level not decrease significantly or even increase after PCI. Although this study didn’t provide direct evidence that Ang-2 of post-PCI can predict the CIN because of the small sample size, we still show highly association of serum Ang-2 levels of post-PCI with the renal-function parameters of post-PCI. Our blood samples were collected within 24 to 48 h after PCI. Further studies need to be done to investigate the relation between the Ang-2 levels of post-PCI in earlier phase and the subsequent occurrence of CIN.

It is well known that CKD patients (even in the lower stages) are more likely to develop CVD than people with a normal kidney function.^[9]^ The classical risk factors sometimes not only fail to predict cardiovascular burden but also show a reverse epidemiology in CKD.^[30]^ Recently, there is growing evidence that one of the principal pathophysiological mechanisms involved in the association of CKD with CVD may be the endothelial dysfunction. The impairment of endothelial function is the initial mechanism that can lead to atherosclerosis.^[31]^ Many risk factors which can affect endothelial function, such as diabetes, obesity, hypertension, and so on, can be found in association with CKD. Besides the common risk factors, the pathophysiologic mechanism of CKD can also directly influence the systemic endothelial functions in multiple ways, such as activation of the renin-angiotensin system, elevation of asymmetric dimethylarginine, increase of circulating cytokines, and so on.^[32]^ Ang-2, which can result in impaired endothelial integrity and function, has recently been shown to possess pro-atherosclerotic effects.^[33]^ Despite that many studies have respectively reported the vital role of Ang-2 in disease of CVD or CKD, the exactly pathophysiologic mechanisms of Ang-2 under the systemic angle are still unclear. Ang-2 may be an important mediator which can enhance systemic vascular burden rather than a simple biomarker of endothelial dysfunction merely reflecting the severity of the disease. Ang-2 is able to sensitize endothelial cells towards TNF-α, which can stimulate the upregulation of related adhesion molecules.^[34]^ These molecules can encourage the migration of monocytes into the arterial wall through the endothelium, promote extracellular matrix degradation and make the plaque vulnerable. It is recently reported that Ang-2 is an independent predictor of major adverse cardiovascular events in CKD patients.^[10]^ In addition, previous studies have shown that the elevated Ang-2 level is an indicator of abnormal cardiac structure and vascular atherosclerotic burden in patients with CKD,^[35,36]^ which supported our hypothesis that the Ang-2 released from 1 dysfunctional organ may accelerate the disease progress of distant organ. On the other hand, in patients with CAD, the Ang-2 released from the heart may also exacerbate the renal function by enhancing the glomerular endothelial apoptosis, aggravating the tissue edema and leading to the albuminuria.^[37]^ Therefore, altered angiopoietin/Tie-2 system may cause a cascade leading to the systemic endothelial dysfunction and subsequent organs dysfunction. However, the exact mechanisms need to be further investigated. Ang-2 may become a novel target to develop therapeutic strategies against CVD or CKD in the future.

Of note, some other mediators have been recently reported probably to be involved in the pathophysiologic mechanism accounting for the high risk of CVD in CKD patients. For instance, microRNAs (miRNAs), the small non-coding RNA molecules that function in RNA silencing and post-transcriptional regulation of gene expression, have been reported to contribute to both the induction and progression of CKD.^[38]^ In addition, circulating miRNAs can be used as diagnostic or prognostic biomarkers in cardiovascular disease, and may have putative function as long-distance communicators enhancing systemic burden.^[39]^ Neopterin, a marker of inflammation and of immune system activation, is synthesized by activated macrophages. After exclusion of patients with known cardiovascular disease, serum neopterin levels are elevated and correlated with the severity of CKD.^[40]^ Besides, there is also a strong association of neopterin with CVD. Studies suggested that it can induce a pro-atherothrombotic phenotype in human coronary endothelial cells and can be used as a biomarker predicting the adverse cardiovascular events.^[41,42]^ Urotensin II (UT-II), which plays an important role in CKD,^[43]^ is involved with endothelial dysfunction and coronary thrombosis.^[44]^ It probably represents a key link between CKD and CAD. It may also have potential to be a biomarker of CIN, since one study showed that serum UT-II levels of post-PCI are significantly correlated with the amount of contrast media in patients undergoing PCI.^[45]^ Oddly, plasma UTN is found to be an inverse predictor of cardiovascular mortality in patients with CKD.^[46]^ Therefore, it will be intriguing to further assess the relationship of Ang-2 with these potential mediators after PCI, and their associations with the occurrence of CIN and subsequent adverse cardiovascular events in the future.

However, there are some limitations to our study. First, because of the small sample size, how the Ang-2 levels will change in CIN patients after PCI, remains to be further investigated. Second, we excluded the patients of STEMI and all of our subjects have no renal failure or uremia, which may underestimate the influence of renal function on serum Ang-2 levels. Therefore, studies on a larger sample size should be performed in the future.

## Author contributions

**Conceptualization:** Chun Gui.

**Data curation:** Wen Jian, Lang Li, Xiao-Min Wei, Chun Gui.

**Formal analysis:** Wen Jian, Lang Li, Jia-Hui Guan, Chun Gui.

**Funding acquisition:** Chun Gui.

**Investigation:** Wen Jian, Xiao-Min Wei, Chun Gui.

**Methodology:** Jia-Hui Guan, Chun Gui.

**Project administration:** Lang Li, Xiao-Min Wei, Chun Gui.

**Resources:** Lang Li, Chun Gui.

**Software:** Wen Jian, Jia-Hui Guan, Guo-Liang Yang, Chun Gui.

**Supervision:** Lang Li, Chun Gui.

**Validation:** Wen Jian, Lang Li, Chun Gui.

**Visualization:** Wen Jian, Lang Li, Chun Gui.

**Writing – original draft:** Wen Jian.

**Writing – review & editing:** Wen Jian, Chun Gui.
